# Associative analysis of multi-omics data indicates that acetylation modification is widely involved in cigarette smoke-induced chronic obstructive pulmonary disease

**DOI:** 10.3389/fmed.2022.1030644

**Published:** 2023-01-12

**Authors:** Junyin Gao, Hongjun Liu, Xiaolin Wang, Liping Wang, Jianjun Gu, Yuxiu Wang, Zhiguang Yang, Yunpeng Liu, Jingjing Yang, Zhibin Cai, Yusheng Shu, Lingfeng Min

**Affiliations:** ^1^Department of Pulmonary and Critical Care Medicine, Northern Jiangsu People's Hospital, Clinical Medical College, Yangzhou University, Yangzhou, China; ^2^Department of Thoracic Surgery, Northern Jiangsu People's Hospital, Clinical Medical College, Yangzhou University, Yangzhou, China; ^3^Clinical Medical College, Yangzhou University, Yangzhou, China; ^4^Department of Cardiology, Institute of Translational Medicine, Clinical Medical College, Yangzhou University, Yangzhou, China; ^5^Department of Thoracic Surgery, The First Hospital of Jilin University, Changchun, China

**Keywords:** COPD, transcriptomics, proteomics, acetylomics, multi-omics associative analysis, single-cell RNA sequencing

## Abstract

We aimed to study the molecular mechanisms of chronic obstructive pulmonary disease (COPD) caused by cigarette smoke more comprehensively and systematically through different perspectives and aspects and to explore the role of protein acetylation modification in COPD. We established the COPD model by exposing C57BL/6J mice to cigarette smoke for 24 weeks, then analyzed the transcriptomics, proteomics, and acetylomics data of mouse lung tissue by RNA sequencing (RNA-seq) and liquid chromatography-tandem mass spectrometry (LC-MS/MS), and associated these omics data through unique algorithms. This study demonstrated that the differentially expressed proteins and acetylation modification in the lung tissue of COPD mice were co-enriched in pathways such as oxidative phosphorylation (OXPHOS) and fatty acid degradation. A total of 19 genes, namely, *ENO3, PFKM, ALDOA, ACTN2, FGG, MYH1, MYH3, MYH8, MYL1, MYLPF, TTN, ACTA1, ATP2A1, CKM, CORO1A, EEF1A2, AKR1B8, MB*, and *STAT1*, were significantly and differentially expressed at all the three levels of transcription, protein, and acetylation modification simultaneously. Then, we assessed the distribution and expression in different cell subpopulations of these 19 genes in the lung tissues of patients with COPD by analyzing data from single-cell RNA sequencing (scRNA-seq). Finally, we carried out the *in vivo* experimental verification using mouse lung tissue through quantitative real-time PCR (qRT-PCR), Western blotting (WB), immunofluorescence (IF), and immunoprecipitation (IP). The results showed that the differential acetylation modifications of mouse lung tissue are widely involved in cigarette smoke-induced COPD. *ALDOA* is significantly downregulated and hyperacetylated in the lung tissues of humans and mice with COPD, which might be a potential biomarker for the diagnosis and/or treatment of COPD.

## 1. Introduction

The 2022 Global Initiative for Chronic Obstructive Lung Disease (COPD) report considered that cigarette smoking is the leading environmental risk factor for COPD (1). The smoke produced by cigarette combustion contains a large number of harmful components (2) that can induce oxidative stress in lung cells (3), cause damage to mitochondria (4, 5), aggravate protease–anti-protease imbalance (6, 7), cause autoimmune response (8, 9), and also cause autophagy dysfunction of lung cells and mitochondria (10–12). These comprehensive factors lead to DNA and protein damage, inflammatory infiltration, cell aging and apoptosis, destruction and remodeling of airway structure, and participate in the progression of COPD.

Liquid chromatography-tandem mass spectrometry (LC-MS/MS) has greatly promoted the determination of post-translational modifications (PTMs) on the protein, such as acetylation (13). Histone acetylation modification leads to chromatin remodeling, regulates the transcriptional activity and gene expression, and is largely independent of the regulation of transcription by DNA methylation (14). Non-histone acetylation modification can regulate RNA transcription, DNA damage repair, and autophagy; alter the structure of the protein, enzyme activity, and signal transduction; effectuate crosstalk on the other types of PTMs, such as phosphorylation; and finally affect the expression and function of proteins (15). Initially, attention was focused on histone proteins with differentially expressed acetylation modifications in COPD (16, 17), and the role of differential acetylation modifications of non-histone proteins induced by cigarette smoke has also attracted attention in recent years (18, 19).

Single-cell RNA sequencing (scRNA-seq) is another emerging technology that has attracted many researchers' attention in recent years and is predicted to have a broader application prospect, including the integration of scRNA-seq data with other omics (scMultiomics) (20). Various advanced high-throughput sequencing technologies have generated several types of omics data. Although single-omics data, such as genomics (21), epigenomics (22), transcriptomics (23, 24), proteomics (25, 26), metabolomics (27, 28), and scRNA-seq (29, 30), have contributed to clarifying the mechanisms of COPD, the disease is still one of the three leading causes of deaths worldwide, and the burden of COPD is expected to increase in the next few decades (1). There is no simple correspondence between transcription and protein abundance; complex regulatory mechanisms affect transcription, translation, PTMs, and metabolic processes, and ultimately affect protein expression (31, 32). Although scRNA-seq can decipher the regulatory correlations among genes from various cell subpopulations and record the trajectories of distinct cell lineages during development, it cannot reveal their spatial distribution or functional characteristics (33). These reasons indicate that a single-omics data set may not be able to fully explain COPD. This finding indicated that there is an urgent need for novel research ideas, such as combining multiple omics data sets. Some studies on COPD have used this strategy (34, 35), indicating that multi-omics data analysis is conducive to identifying biomarkers and understanding the heterogeneity of COPD. Li et al. showed that integrating multiple omics data improves the accuracy of diagnosis and molecular subtype prediction of COPD compared with single-omics data (36). In addition, Lai et al. (37) and Pei et al. (38) correlated scRNA-seq with other omics data to study the genetic characteristics and pathogenesis of COPD.

In this study, we aimed to elucidate the molecular mechanisms underlying lung injury in COPD mice at the levels of gene transcription, protein translation, and PTMs through multi-omics data associative analysis; reveal the role of protein acetylation modification in COPD; and discover new potential prevention and treatment targets of COPD. Also, we analyzed the distribution of these genes in cell subpopulations of lung tissue by scRNA-seq in order to select the appropriate genes for subsequent experimental verification and lay a foundation for further studies on the pathway mechanisms of COPD in the future.

## 2. Materials and methods

### 2.1. Animals

In this study, six male specific pathogen-free (SPF) grade C57BL/6J mice, 6 weeks old, were purchased from Charles River (CRL) experimental Animal Center (Beijing, China; License key: SCXK, Beijing, 2016-0006). After 2 weeks of adaptive feeding, the animals were randomly and equally divided into the control group (control) and the cigarette smoke treatment group (CS). Mice in the CS group were exposed to the smoke of 3R4F research cigarettes (Tobacco Research Institute, University of Kentucky, Lexington, KY; 11 mg TPM, 9.4 mg tar, and 0.73 mg nicotine per cigarette), 5 cigarettes and 30 min one time, twice a day, at an interval of 4 h, 5 days/week for 24 weeks, while the mice in the control group were free to breathe fresh air. During the experiment, mice ate and drank freely in a controlled environment: 12:12 h of light:dark cycle, a humidity of 50–60%, and a temperature of 21–23°C. During the whole experiment, the animals were given humanitarian care in accordance with the 3R principle. The study was approved by the ethics committee of the Clinical Medical College of Yangzhou University (Yangzhou, Jiangsu Province, China).

### 2.2. Lung function measurement

The lung function of the two groups of mice was measured by a forced oscillatory small-animal ventilator (flexiVent, SCIREQ) in the Function Experiment Center of the Clinical Medical College of Yangzhou University. To ensure the accuracy of data, the measurement process was operated by the same person. That is, the mice were anesthetized before tracheostomy, and the endotracheal intubation was connected with the small-animal ventilator. The parameters were as follows: performed quasi sinusoidal ventilation, tidal volume: 10 ml/kg, respiratory rate: 150 times/min, I:E ratio: 2:3, and PEEP: 3 cmH_2_O. Then, the airway resistance (RN), tissue damping (G), tissue elasticity (H), peak expiratory flow (PEF), forced expiratory volume in 100 ms (FEV0.1), forced vital capacity (FVC), and FEV0.1/FVC ratio were measured and recorded. Each mouse was tested repeatedly six times, and the average value of each item was considered.

### 2.3. Histopathological analysis and morphometry

The left lower lung tissue of mice was fixed with 4% formaldehyde for 24 h, embedded in paraffin after dehydration, sectioned (5-μm thick, hematoxylin and eosin [H&E]), and stained with H&E after dewaxing and rehydration. The stained sections were observed, evaluated, and photographed by experienced pathological researchers at 100× and 400× magnification under the optical microscope (OLYMPUS BX53, with the image analysis software Stream). The Image-Pro Plus (Media Cybernetics, Rockville, MD, USA) was used to analyze the images, and the mean linear intercept (MLI) was measured: six images from different shooting fields of each sample (did not contain airways and/or blood vessels) were overlaid with an 11-horizontal line template. The intercepts of the alveolar walls with lines were enumerated, and then the total length of the 11 lines was divided by the average number of intercepts (39).

### 2.4. Transcriptomics

According to the manufacturer's instructions, total RNA was extracted and purified from the left upper lung tissue of each mouse and amplified by polymerase chain reaction (PCR). The constructed library was examined on an Agilent 2100 Bioanalyzer and ABI StepOneplus Real-Time PCR System and sequenced on the Illumina HiSeq platform. The clean reads were obtained by removing low-quality reads, adapters, and poly-N sequences from the raw reads. The clean reads were matched to the reference genome sequence (GRCm38) using HISAT2-software, new transcripts were predicted, and single-nucleotide polymorphism (SNP), insertion-deletion (InDel), and differential splicing genes (DSG) were identified. The new transcript with protein coding potential was added to the reference gene sequence to form a complete reference sequence and then the gene expression was calculated. Finally, the quality of data from each sample and the differentially expressed genes between different samples were analyzed (40). Due to a large amount of transcriptome differential analysis data, Benjamini–Hochberg adjustment was performed on *p*-value to further reduce the false-positive rate. Subsequently, we defined genes with more than a 2-fold difference and *p* < 0.001 after correction as significantly differentially expressed genes.

### 2.5. Proteomics

An appropriate amount of the right lower lung tissue of each mouse was ground and homogenized, and the supernatant was collected by centrifugation to determine the protein concentration using the BCA method. After trypsin digestion, the peptide was desalted on the Strata X C18 SPE column (Phenomenex) and vacuum dried. The peptide was reconstituted in 0.5 M TEAB and processed using the TMT kit, according to the manufacturer's protocol. The tryptic peptides were fractionated by high pH reverse-phase HPLC and separated on a gradient of 8–32% acetonitrile (pH 9.0) over 60 min into 60 fractions; these were pooled into 18 fractions and dried by vacuum centrifugation.

For LC-MS/MS analysis, the tryptic peptides were solubilized in solvent A (aqueous solution containing 0.1% formic acid and 2% acetonitrile). The gradient comprised an increase from 9 to 23% of solvent B (aqueous solution containing 0.1% formic acid and 90% acetonitrile) in 0–26 min, from 23 to 35% in 26–34 min, and 23 to 80% in 34–37 min, then held at 80% for the last 3 min; a constant flow rate of 300 nl/min was maintained on the EASY-nLC 1000 UPLC system throughout these processes. The peptides were subjected to an NSI source, followed by Orbitrap Fusion mass spectrometry. The electrospray voltage was set at 2.0 kV. The scanning range of primary mass spectrometry was set to 350–1,550 m/z, and the scanning resolution was set to 60,000. The scanning range of the secondary mass spectrometry was fixed at 100 m/z, and the secondary scanning resolution was set at 30,000. The DDA program was selected as the data acquisition mode. The automatic gain control (AGC) was set at 5E4 to improve the effective utilization of mass spectrometry. The signal threshold was set to 5,000 ions/s, and the maximum injection time was set to 100 ms. To avoid repeated scanning, a data-dependent procedure was alternated between the scans with dynamic exclusion of 30 s.

Maxquant search engine (version 1.5.2.8) was used to process the resulting data searched against the mouse SwissPort database concatenated with the reverse decoy database to calculate the false discovery rate (FDR) caused by random matching, and common contamination databases were added to eliminate the influence of contaminated proteins. Trypsin/P was specified as the cleavage enzyme allowing up to two missing cleavages. The minimum length of the peptide was set to seven amino acid residues. The mass tolerance for precursor ions was set at 20 ppm in the first search and 5 ppm in the main search and was set for fragment ions at 0.02 Da. The quantitative method was set to TMT-10plex, and FDR was adjusted to <1%. It was defined as a significantly differentially expressed protein if the ratio of change was > 1.3 or <1/1.3 and *p* < 0.05.

### 2.6. Acetylomics

The processes of protein extraction, trypsin digestion, and TMT labeling were consistent with proteomics. HPLC fractionation was also similar to proteomics, except that peptides were combined into 4, but not 18, fractions before being dried by vacuum centrifugation. In addition, there was an additional process of affinity enrichment between HPLC fractionation and LC-MS/MS analysis. To enrich the acetylated peptides, tryptic peptides dissolved in NETN buffer (100 mM NaCl, 1 mM EDTA, 50 mM Tris–HCl, 0.5% NP-40, pH 8.0) were incubated with pre-washed antibody beads at 4°C overnight, with gentle shaking. Then, the beads were washed four times with NETN buffer and twice with deionized water. The bound peptides were eluted from the beads with 0.1% trifluoroacetic acid. Finally, the eluted fractions were combined and vacuum-dried. The resulting peptides were desalted with C18 ZipTips (Millipore) before LC-MS/MS analysis, according to the manufacturer's instructions. The processes of LC-MS/MS analysis and database search were similar to those described in proteomics, except that there were only a very few different parameter settings. Similarly, we defined significantly differentially expressed protein acetylation modification according to the standard of the ratio of change > 1.3 or <1/1.3 and *p* < 0.05.

### 2.7. Multi-omics associative analysis

#### 2.7.1. Transcriptomics and proteomics associative analysis

##### 2.7.1.1. Protein annotation

First, we screened the proteins quantified at both transcriptome and proteome levels. The protein ID was converted to UniProt ID, and the GO ID was matched with UniProt ID in order to obtain the corresponding information from the UniProt-GOA database (http://www.ebi.ac.uk/GOA/) for Gene Ontology (GO) annotation, according to GO ID. If no protein information was queried in the database, an algorithm software based on protein sequence—InterProScan (version 5.14-53.0, http://www.ebi.ac.uk/interpro/)—would be used to predict the GO function of the protein. Next, the Kyoto Encyclopedia of Genes and Genomes (KEGG) online service tool KAAS (version 2.0, http://www.genome.jp/kaas-bin/kaas_main) was used to annotate the screened proteins and matched into the corresponding pathways in the database by KEGG mapper (version 2.5, http://www.kegg.jp/kegg/mapper.html).

##### 2.7.1.2. Protein functional enrichment

All the differential expressions on at least one level of transcriptome and proteome were screened and divided into groups according to different expression trend types in the transcriptome and proteome (up-up, up-down, up-unchanged, down-unchanged, down-down, down-up, unchanged-up, and unchanged-down) to study the potential correlation between different regulatory relationships and specific functions. Briefly, GO functional enrichment was performed separately with the differentially expressed genes and proteins in various groups and classified into three categories: cellular compartment (C-C), biological process (B-P), and molecular function (M-F). Similarly, the KEGG database was used to identify the enriched pathways for different groups. The two-tailed Fisher's exact test (Perl module, version 1.31, https://metacpan.org/pod/Text::NSP::Measures::2D::Fisher) was used in the enrichment test; *p* < 0.05 was considered significant.

##### 2.7.1.3. Enrichment-based clustering

All the functional enrichments and pathways that were significant (*p* < 0.05) in at least one regulatory relationship group were screened. The filtered *p*-value data matrix was first transformed by a logarithm of -log10; then, the transformed data matrix was converted by *z*-transformation. These *z* scores were clustered by one-way hierarchical clustering (Euclidean distance, average linkage clustering) in Genesis. The cluster membership was visualized by heat maps using the heatmap (R package, https://cran.r-project.org/web/packages/cluster/).

#### 2.7.2. Proteomics and acetylomics associative analysis

##### 2.7.2.1. Acetylomics analysis

All the proteins corresponding to the differentially expressed acetylation modification sites were annotated and functionally enriched with GO and KEGG; InterPro domain database (http://www.ebi.ac.uk/interpro/) was used to annotate the protein domains, then enrichment-based clustering was performed using the methods described earlier. The subcellular localization was annotated by the Wolfpsort software (version 0.2, http://www.genscript.com/psort/wolf_psort.html). Soft MoMo (motif-x algorithm, version 5.0.2, http://meme-suite.org/tools/momo) was used for motif analysis to analyze the motif characteristics of the modification sites.

##### 2.7.2.2. Proteomics and acetylomics associative analysis

All the identifiers of differentially expressed proteins and proteins corresponding to the differentially expressed acetylation sites (defined as proteome and modification, respectively) were collected. These proteins were searched against the STRING database version 11.0 for protein–protein interactions. Only interactions between the proteins belonging to the searched data set were selected, and the external candidates were excluded. STRING defines a metric termed “confidence score” to define the interaction confidence. Next, we fetched all interactions with a confidence score of ≥ 0.7 (high confidence) and defined the number of proteins interacting with other differentially expressed proteins as “degree.” The interaction network forming STRING was visualized in the Cytoscape software, and a graph theoretical clustering algorithm—molecular complex detection (MCODE, plugin of Cytoscape)—was utilized to analyze the densely connected regions (proteins with the highest “degree”). Finally, we carried out functional enrichment on most proteins in these densely connected regions (marked by dotted circles).

### 2.8. Single-cell RNA sequencing (scRNA-seq) data analysis

The scRNA-seq data (GSE136831) of whole lungs dissociated from *Homo sapiens* were downloaded from the National Center of Biotechnology Information (NCBI) Gene Expression Omnibus (GEO; https://www.ncbi.nlm.nih.gov/geo/). To analyze accurately, we selected eight samples of patients with COPD as the COPD group and eight samples of healthy humans as the control group from GSE136831 (41). These samples were age and sex matched, and at least 1,500 cells were detected in each sample. The quality control standard was that the cells expressed at least 500 and at most 8,000 genes, the proportion of mitochondria was <20%, and the total RNA detected was <80,000, following which the doublets generated due to the excessive number of genes and RNA detection were removed. The data were standardized and scaled using the Seurat R package. The top 2,000 highly variable genes and the top 10 significant PCs were used to perform the dimension reduction cluster analysis downstream. The cell subpopulations in lung tissue were identified based on the expression of classical correlative biomarkers. The genes from various cell subpopulations with the absolute value of the differential expression (log2) between the two groups > 0.25 were defined as significantly differentially expressed genes (*p* < 0.05).

### 2.9. Quantitative real-time PCR (qRT-PCR)

RNAsimple total RNA kit (DP419; Tiangen Biotech Co., Ltd., Beijing, China) was used to extract the RNA from a part of the right upper lung tissue of mice. The cDNA was generated from 0.4 μg of RNA using the HiScript III RT SuperMix for qPCR (+gDNA wiper; R323-01; Vazyme Biotech Co., Ltd., Nanjing, Jiangsu, China). Next, we used AceQ^®^ Universal SYBR^®^ qPCR Master Mix (Q511-02; Vazyme Biotech Co., Ltd.) and ABI Step One Plus real-time fluorescence quantitative PCR instrument (Applied Biosystems, Foster City, CA, USA) for qRT-PCR. *GAPDH* was used as the internal normalization control, and the difference between the two groups was analyzed using the 2^−ΔΔCt^ method. The primer sequences were as follows: *ALDOA*-mouse, forward: 5′-GGAACCAATGGCGAGACAACTACC-3′, reverse: 5′-GGCAAAGTCGGCTCCATCCTTC-3′; and *GAPDH*-mouse: forward: 5′-GGCAAATTCAACGGCACAGTCAAG-3′, reverse: 5′-TCGCTCCTGGAAGATGGTGATGG-3′.

### 2.10. Western blotting (WB) analysis

RIPA lysis buffer (P0013B; Beyotime Biotechnology, Shanghai, China) containing phenylmethanesulfonylfluoride (P0100; Solarbio Science & Technology Co., Ltd., Beijing, China) was used to extract the total protein from the remaining right upper lung tissue of mice. The supernatant was collected after centrifugation at 4°C at 12,000 rpm for 15 min, and the protein concentrations were determined by a BCA protein quantification kit (E112-02; Vazyme Biotech Co., Ltd.). The protein was denatured by loading buffer (FD002; Fude Biological Technology Co., Ltd., Hangzhou, Zhejiang, China) and separated by electrophoresis on 10% SDS-PAGE (E303-01; Vazyme Biotech Co., Ltd.) and transferred to 0.2-μm polyvinylidene fluoride membranes (ISEQ00010; Merck Millipore, Billerica, MA, USA). The membranes were blocked in Quick Block Liquid (P0252; Beyotime Biotechnology) at room temperature for 15 min. The block liquid was eluted with Tris-buffered saline containing 0.1% Tween 20 (v/v; TBST). Subsequently, the membranes were probed with primary antibodies (*ALDOA* rabbit polyclonal antibody, AF6189, 1:10,000 dilution; beta-tubulin rabbit monoclonal antibody, AF1216, 1:2,000 dilution; Beyotime Biotechnology) at 4°C overnight and incubated with the corresponding secondary antibody [horseradish peroxidase-labeled goat anti-rabbit IgG (H+L), A0208, 1:5,000 dilution; Beyotime Biotechnology] at room temperature for 2 h after elution with TBST. The immunoreactive bands were visualized using the hypersensitive ECL chemiluminescence kit (P0018S; Beyotime Biotechnology) and quantified on a Bio-Rad ChemiDoc XRS+ chemiluminescence gel imaging system with the Image Lab software (Bio-Rad Laboratories, Hercules, CA, USA).

### 2.11. Immunofluorescence (IF) staining

The paraffin blocks were sectioned and dewaxed. After antigen retrieval, the sections were incubated by primary antibody (*ALDOA* polyclonal antibody, 11217-1-AP, 1:200 dilution; Proteintech, Wuhan, Hubei, China) at 4°C overnight, washed three times with phosphate-buffered saline (PBS), and incubated with the secondary antibody [Goat anti-rabbit IgG (H+L) Fluor594-conjugated, S0006, 1:500 dilution; Affinity Biosciences] at room temperature, in the dark, for 1 h. Washed 3 times with PBS, the nuclei were stained using a DAPI staining kit (KGA215; KeyGEN BioTECH Co., Ltd., Nanjing, Jiangsu, China) at room temperature, in the dark, for 5 min. Next, the coverslips were mounted with an antifade mounting solution (Invitrogen, Carlsbad, CA, USA). Finally, sections were scanned with a digital pathological section scanner (Olympus VS200, Japan). The OlyVIA software was used to analyze the observed sections and randomly capture six images of non-overlapping visual fields at 200 × magnification from each sample. The fluorescence intensity of the positively stained cells was evaluated with the Image J software (National Institutes of Health).

### 2.12. Immunoprecipitation (IP)

IP kit (P2197M; Beyotime Biotechnology) was used to carry out the IP experiments according to the manufacturer's instructions. The protein was extracted from the lung tissue of mice with a lysis buffer containing protease inhibitor and deacetylase inhibitor (P1112; Beyotime Biotechnology). An appropriate amount of protein A+G agarose gel beads was incubated with the indicated antibody (11217-1-AP; Proteintech) at 50 μg/ml at room temperature for 1 h. Subsequently, the protein samples were added to the mixture and incubated at 4°C overnight. The supernatant was discarded, and the agarose beads were washed on ice with RIPA lysis buffer. The resulting immunoprecipitated complexes were denatured by loading buffer and analyzed by immunoblotting (primary antibody: pan acetyl-lysine rabbit polyclonal antibody, AF5632, 1:1,000 dilution; Beyotime Biotechnology; secondary antibody: A0208, 1:5,000 dilution; Beyotime Biotechnology). Next, the expression of *ALDOA* was visualized, and the immunoreactive bands were analyzed as described earlier.

### 2.13. Statistical analysis

qRT-PCR, WB, and IP were repeated at least three times. All data were processed using the SPSS software, version 23.0 (Chicago, IL, USA), and the results are presented as mean ± standard error of the mean (mean ± SEM). Two-group comparisons were analyzed using the Student's *t*-test. Wilcoxon rank-sum test was used to compare the results between the two groups and the Kruskal-Wallis test to determine the difference among groups. A *p*-value <0.05 indicated a statistically significant difference.

## 3. Results

### 3.1. The lung function of mice treated with cigarette smoke was impaired

**Figure 1:**
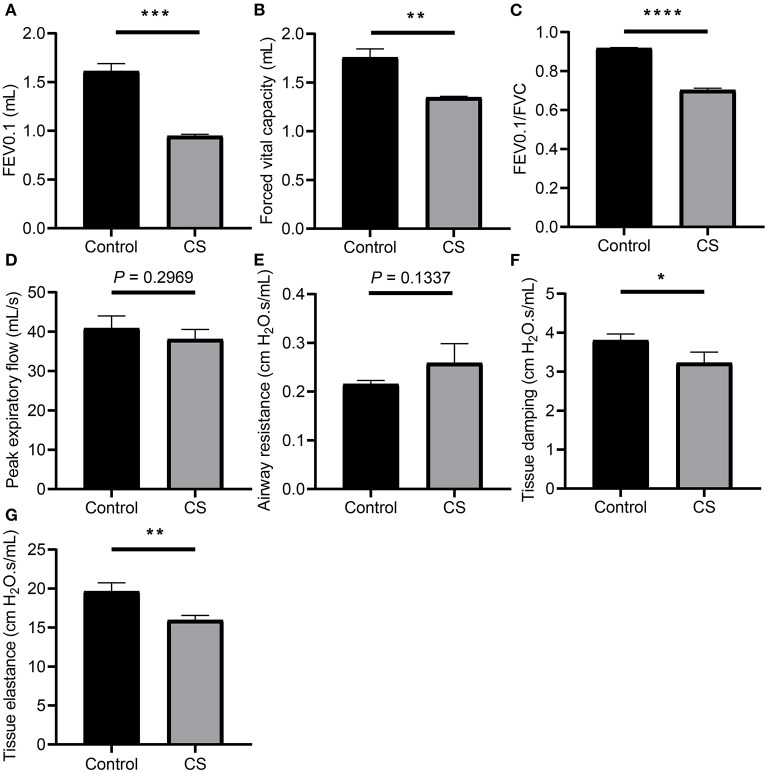
Lung function measurement of mice from cigarette smoke treatment group and control group. **(A)** Forced expiratory volume in 100 ms (FEV0.1). **(B)** Forced vital capacity (FVC). **(C)** Ratio of FEV_1_0.1 to FVC. **(D)** Peak expiratory flow (PEF). **(E)** Airway resistance (Rn). **(F)** Tissue damping **(G)**. **(G)** Tissue elastance **(H)**. *p < 0.05, **p < 0.01, ***p < 0.001, and ****p < 0.0001.

The results of the pulmonary function test showed that FEV0.1 (1.611 ± 0.0447 ml, 0.9469 ± 0.0097 ml, *p* = 0.0001), FVC (1.757 ± 0.0514 ml, 1.349 ± 0.005 ml, *p* = 0.0014), and FEV0.1/FVC ratio (0.9174 ± 0.0016, 0.7023 ± 0.0056, *p* < 0.0001) decreased significantly in the CS group, while the differences in PEF (40.86 ± 1.815 ml/s, 38.1 ± 1.428 ml/s, *p* = 0.2969) and airway resistance (Rn; 0.2153 ± 0.0043 cmH_2_O.s/ml, 0.2589 ± 0.0228 cmH_2_O.s/ml, *p* = 0.1337) were not significant, the tissue damping (G; 3.804 ± 0.0927 cmH_2_O.s/ml, 3.227 ± 0.1582 cmH_2_O.s/ml, *p* = 0.0345), and tissue elastance (19.65 ± 0.6328 cmH_2_O.s/ml, 15.97 ± 0.3311 cmH_2_O.s/ml, *p* = 0.0068) of the CS group decreased significantly, indicating airflow restriction and impairment of lung tissue elasticity in the CS group (Figure 1, Supplementary Table 1).

### 3.2. Histopathological changes of lung tissue in mice treated with cigarette smoke

**Figure 2:**
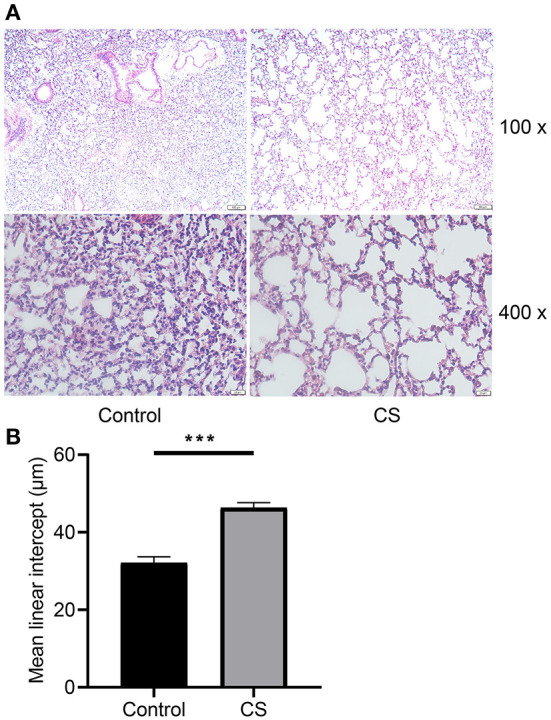
Histopathological analysis and morphometry of mouse lung tissues from cigarette smoke treatment group and control group. **(A)** Hematoxylin–eosin staining of mouse lung tissues from two groups. **(B)** Mean linear intercept of mice from two groups. ***p < 0.001.

H&E staining showed that the alveolar cavities were larger, part of the alveolar septa were broken, alveolar cavities were fused, emphysema was formed (Figure 2A), and the MLI was significantly larger in the lung tissue of mice treated with cigarette smoke than in the control group (32.12 ± 0.8859 μm, 46.3 ± 0.7706 μm, *p* = 0.0003; Figure 2B).

### 3.3. Multi-omics associative analysis

#### 3.3.1. Transcriptomics and proteomics associative analysis

**Figure 3:**
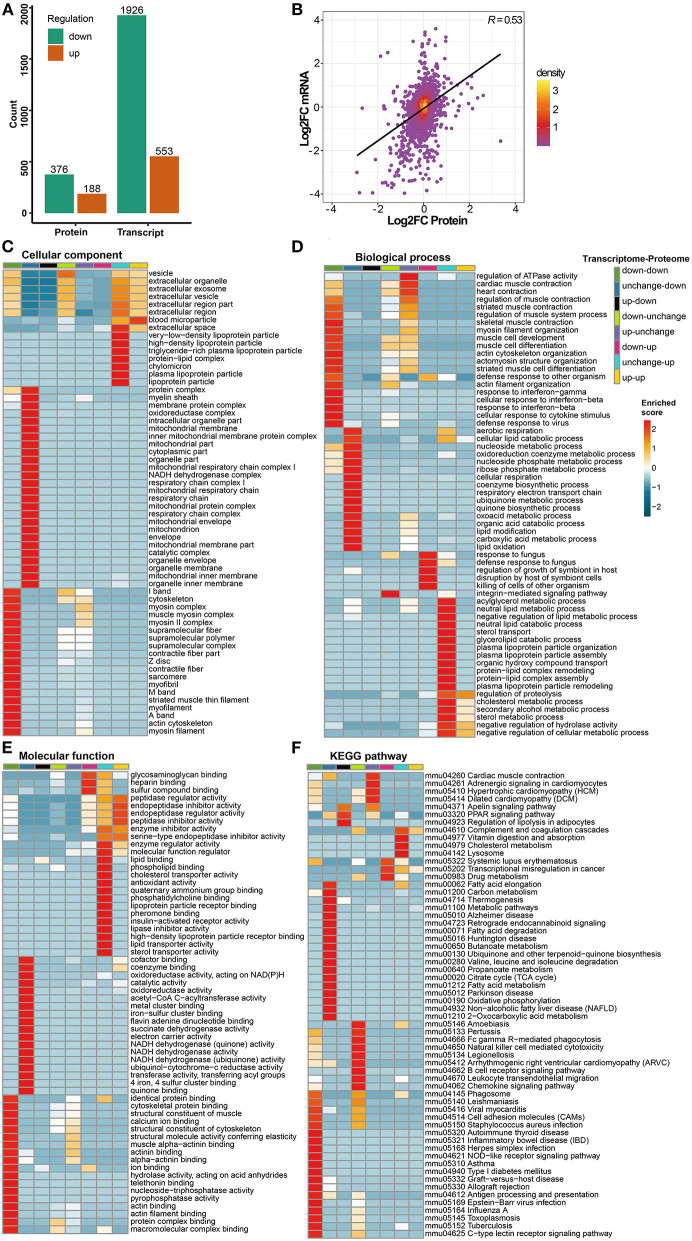
Transcriptomics and proteomics associative analysis. **(A)** Number of differentially expressed genes and proteins in transcriptome and proteome. Green means significantly downregulated genes and proteins, orange means significantly upregulated genes and proteins. **(B)** Scatterplot of transcript and its corresponding protein expression. The horizontal axis shows the protein expression and the vertical axis shows the transcript expression (convert to log2 ratio format), the color of the point indicates the density of the point, “*R*” means the correlation degree between transcriptome and proteome. **(C**–**E)** Transcriptomics and proteomics associative analysis based on GO functional enrichment. The color of the heat map indicates the *p*-value transformed by log10 and Z score, the redder color indicates that the enrichment is more significant. **(F)** Transcriptomics and proteomics associative analysis based on KEGG pathway enrichment. Similarly, the color of the heat map indicates the *p*-value transformed by log10 and *z* score, and the redder color indicates the more significant enrichment.

A total of 23,024 transcripts and 6,153 proteins were quantified in the transcriptome and proteome, respectively. Out of these, 5,822 genes were quantified at both the transcriptome and proteome levels. Compared with the control group, 2,479 differentially expressed genes were defined in the transcriptome of the cigarette smoke treatment group, of which 553 were upregulated and 1,926 were downregulated. A total of 564 differentially expressed proteins were defined in proteomics, of which 188 were upregulated and 376 were downregulated (Figure 3A). As mentioned previously, genes and proteins differentially expressed in at least one omics were subdivided into eight groups according to the different trends (Supplementary Table 2). Among these, only 162 genes were differentially expressed with consistent trends at both the transcriptome and proteome levels (135 down-down, 27 up-up) and listed in Table 1 together with the number of genes in each group. The expression of genes quantified in both transcriptome and proteome was combined, drawn into a scatter diagram, and the correlation between the two omics data was calculated (*R* = 0.53; Figure 3B).

**Table 1:** Grouping according to types of difference trends between transcriptomics and proteomics.

**Group**	**Number of genes**	**Gene name**
Down-down	135	*ALDOA, CYTH4, PADI2, TPM1, IFIT3, PTPN6, TRIM34A, MFAP5, SLC2A4, LPXN, IKZF1, SPL10, B2M, SP100, STAT2, H2-Q7, BVL, BST2, LSP1, TREX1, S100A4, CORO1A, RNF213, ADSSL1, ACTN2, AK1, BIN1, PYCARD, STEAP4, ASPN, PLD4, PTPRC, ITGAL, CAP3, MYO1G, DOCK2, CD74, MNDAL, FMOD, PFKM, TMEM38A, TAP1, MPZ, PYHIN1, SRL, SEPT1, SBSN, SH3BGR, H2-AB1, H2-AA, CD48, TRIM72, SMYD1, HSPB6, MB, MYOM1, IRGM1, H2-EB1, TUBB2B, THY1, CSRP3, OBSCN, IFIT1, FSCN1, STFA1, PDLIM3, OAS1A, CALML3, SERPINB2, CD274, TOP2A, OAS3, STAT1, PGAM2, GVIN1, S100A14, FLNC, GBP2, LDB3, ANO5, EEF1A2, TPM2, MNDA, TTN, CMYA5, ZBP1, ABCB4, GZMA, ADA, ENO3, MYOM3, GBP5, IIGP1, TGTP1, PYGM, MZB1, MYBPH, CMA1, ASPRV1, LY6D, FHL3, GBP4, HIST1H1A, MYH3, PRG2, KLHL41, MYL1, SYPL2, APOBEC2, CKM, RPTN, KRT14, SERPINB12, CASQ1, ACTN3, PKP1, SERPINB5, ATP2A1, MYL3, MYBPC2, TGM3, TNNT3, MYLPF, MYOZ1, LGALS7, TNNI2, PVALB, MYOT, MYH4, SPRR3, TNNC2, ACTA1, MYH1, MYH8*, and *CALM4*
Unchanged-down	186	
Up-down	5	
Down-unchanged	1,782	
Up-unchanged	521	
Down-up	10	
Unchanged-up	141	
Up-up	27	*FGA, AGT, PMVK, FGG, SERPINA3K, GCLC, FKBP5, PLAT, ORM1, SEC14L4, TXNRD1, HMOX1, AKR1B8, AHSG, LPL, SERPINA3N, CBR3, LYVE1, TIMP3, QSOX1, LSS, SFTPD, TCN2, ATP7B, TINAGL1, MMP3*, and *SERPINA3M*

Numbers of genes in each group and genes with consistent differential expression trends (down-down, up-up) were shown in this table. Specific genes in other 6 groups with inconsistent differential expression trends are listed in Supplementary Table 2.

In GO functional enrichment, the differential expression of transcriptomics and proteomics was mainly concentrated in groups of unchanged-down, down-down, and unchanged-up (Figures 3C–E). In the C-C dimension, differentially expressed genes and proteins were enriched in organelle components, such as mitochondria, especially the respiratory chain, in the unchanged-down group; the down-down group was mainly enriched in the components of skeletal muscle; the unchanged-up group was mainly enriched in lipoprotein particles and extracellular space (Figure 3C). In the B-P dimension, differentially expressed genes and proteins in the unchanged-down group were mainly enriched in the BPs related to the respiratory electron transport chain (ETC) and lipid metabolism; in response to infection, development, and differentiation of skeletal muscle in the down-down group; and in the biological processes related to lipid metabolism in the unchanged-up group (Figure 3D). In the M-F dimension, the differential expression was mainly enriched in the activity of respiratory chain-related enzymes and binding of related factors in the unchanged-down group; the binding of skeletal muscle-related factors in the down-down group; and in the activity of lipid metabolism-related enzymes and binding of related factors in the unchanged-up group. Interestingly, we enriched the pathways related to the activity and binding of peptidase and endopeptidase in the down-up group, which were related to proteolysis (Figure 3E).

In KEGG pathway enrichment, the differential expression of transcriptomics and proteomics were mainly concentrated in the down-down, unchanged-down, and down-unchanged groups (Figure 3F). In the down-down and down-unchanged groups, the pathways related to inflammation and immunity were mainly enriched, and in the unchanged-down group, the pathways related to fatty acid and amino acid metabolism, mitochondrial respiratory electron chain, and nervous system diseases were enriched. Additionally, we enriched the PPAR signaling pathway in the up-down group, the hypertrophic cardiomyopathy (HCM) in the up-unchanged group, the transcriptional dysregulation in cancer in the down-up group, and the complement and coagulation cascade in the unchanged-up group.

#### 3.3.2. Acetylomics analysis

**Figure 4:**
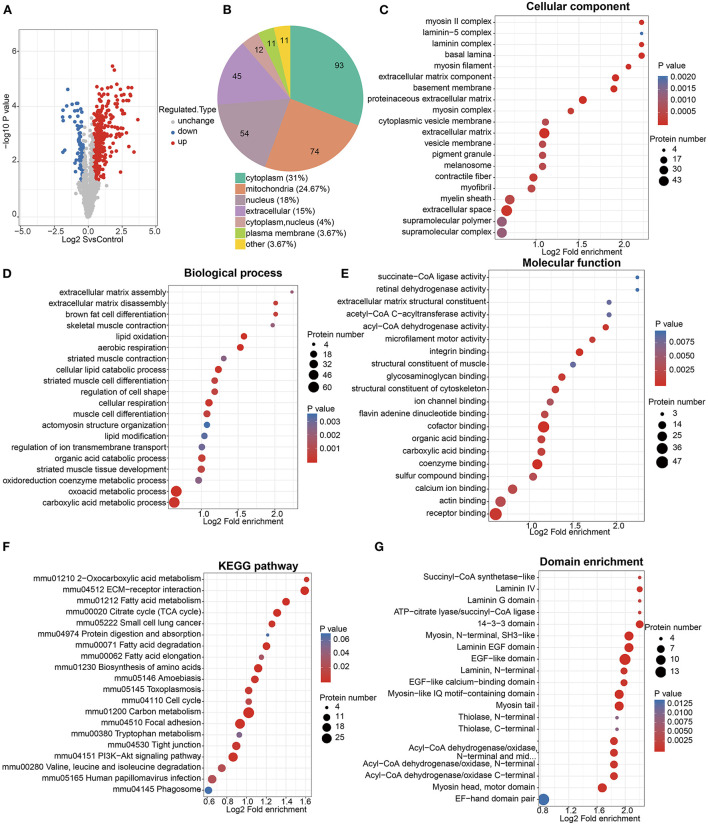
Acetylomics analysis. **(A)** Volcano map of acetylation modification sites differentially expressed between cigarette smoke treatment group and control group. The horizontal axis represents the differential expression between two groups (log2 format), and the vertical axis represents the significance of differential expression (*p*-value, log10 format), red dots represent significantly upregulated modification sites, while blue dots represent significantly downregulated modification sites. **(B)** Subcellular structural localization of proteins corresponding to differential acetylation modification sites. **(C**–**E)** GO functional enrichment of proteins corresponding to differential acetylation modification sites. The size of the circle represents the number of enriched proteins, and the color of the circle represents the significance of enrichment (*p*-value). **(F)** KEGG pathway enrichment of proteins corresponding to differential acetylation modification sites. The size of the circle represents the number of enriched proteins in each pathway, and the color of the circle represents the significance of enrichment (*p*-value). **(G)** Protein domain enrichment of proteins corresponding to differential acetylation modification sites. Similarly, the size of the circle represents the number of enriched proteins in each domain, and the color of the circle represents the significance of enrichment (*p*-value).

Among all the quantified proteins and acetylation modification sites, acetylomics identified 444 upregulated sites of 255 proteins and 89 downregulated sites of 62 proteins in the cigarette smoke treatment group compared with the control group (Figure 4A, Supplementary Table 3). The subcellular structure localization of proteins corresponding to differentially expressed acetylation modification sites was mainly the cytoplasm, mitochondria, nucleus, and extracellular (Figure 4B).

The GO functional enrichment showed that proteins corresponding to differential acetylation sites were mainly enriched in the cellular components such as extracellular space, extracellular matrix (ECM), basement membrane, contractile fiber, and myofibril; the biological processes related to fatty acid metabolism, cell respiration, and skeletal muscle; and the molecular functions of the bindings related to fatty acid metabolism and the enzymatic activity of acyl-CoA dehydrogenase (Figures 4C–E).

The KEGG pathway enrichment (Figure 4F) was mainly enriched in the citrate cycle (TCA cycle), fatty acid metabolism, amino acid metabolism, ECM-receptor interaction, focal adhesion, tight junction, and the Pl3k-Akt signaling pathway.

In addition, the protein domain enrichment of proteins corresponding to differential acetylation sites was mainly focused on laminin, epidermal growth factor (EGF), myosin, and acyl-CoA (Figure 4G).

#### 3.3.3. Proteomics and acetylomics associative analysis

**Figure 5:**
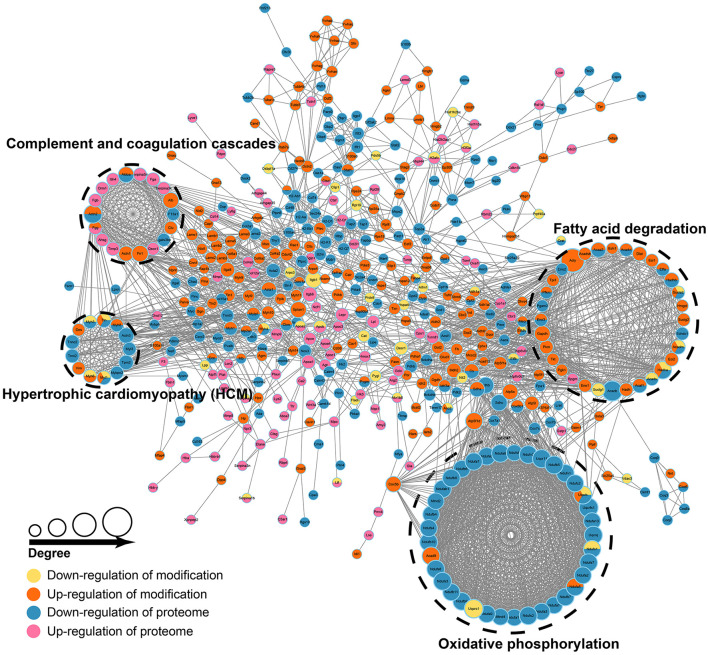
Proteomics and acetylomics associative analysis. The size of the circle represents the value of degree (number of proteins interacting with differentially expressed proteins), the larger the circle, the higher the degree. The colors of the circle represent up/downregulation in proteome and modification.

As described earlier, we collected 508 proteins and annotated them, proteins with high interaction confidence and “degree” were mainly enriched in pathways, such as oxidative phosphorylation (OXPHOS), fatty acid degradation, complement and coagulation cascade, and HCM (Figure 5, Supplementary Table 4). Interestingly, these four pathways were also significantly enriched in the associative analysis of transcriptomics and proteomics. Then, we intersected the 508 proteins with transcriptomics and proteomics data, and 19 genes were differentially expressed in transcriptomics, proteomics, and acetylomics simultaneously. In all, nine genes, namely, *ENO3, PFKM, ALDOA, ACTN2, FGG, MYH3, MYH8, MYL1*, and *TTN*, were involved in these four pathways, while the other 10 genes, namely, *ACTA1, ATP2A1, CKM, CORO1A, EEF1A2, AKR1B8, MB, MYH1, MYLPF*, and *STAT1*, were not involved in either of the pathways. Moreover, the associative analysis identified 35 genes differentially expressed in proteomics and acetylomics, but not in transcriptomics; of these, *NDUFS1, NDUFA5, SDHA*, and *UQCRB* were enriched in the OXPHOS pathway, while *ACADV1, ETFA, HADHB, SUCLA2, ACADM, HADHA, ACADS, ACAT1*, and *ACAA2* were enriched in the fatty acid degradation pathway.

### 3.4. Single-cell RNA sequencing (scRNA-seq) analysis

**Figure 6:**
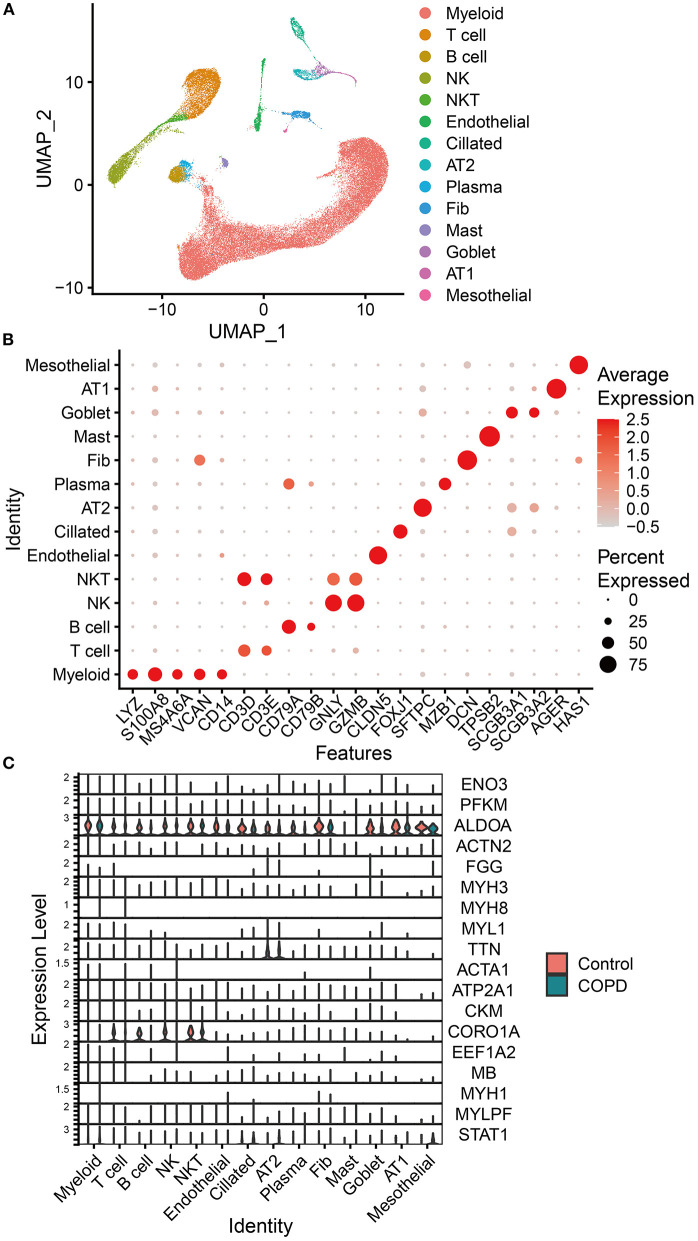
Analysis of scRNA-seq analysis data. **(A)** Uniform manifold approximation and projection (UMAP) of the distribution of different cell subpopulations. **(B)** Expression of classical biomarkers related to 14 cell subpopulations. **(C)** Distribution and expression of the 19 genes identified by associative analysis of multi-omics data in 14 cell subpopulations identified by analyzing scRNA-seq data of lung tissue from COPD patients and healthy humans. The orange violin plots represent the control group of healthy humans, and the green ones represent the group of patients with COPD. The height of violin plots represents the expression of genes, while the width represents the proportion of cells expressing these genes in cell subpopulations.

According to the expression of representative biomarkers, we identified 14 cell subpopulations from scRNA-seq data (GSE136831; Figures 6A, B) and analyzed the distribution of the 19 genes mentioned in this data set (Supplementary Table 5). Among these genes, *AKR1B8* is not expressed in *Homo sapiens*. The results showed that only *ALDOA* and *CORO1A* were differentially expressed in the lungs of COPD patients, which was consistent with the trend of the difference (downregulated) of our transcriptome data. In addition, both genes were downregulated in proteomics and upregulated in acetylomics. *ALDOA* was widely expressed in various cell subpopulations and downregulated in alveolar type 2 cells (AT2), goblet cells, B lymphocytes (B cell), natural killer cells (NK cells), and fibroblasts (Fibs; Figure 6C).

### 3.5. *ALDOA* was downregulated and hyperacetylated in the lung tissue of COPD mice

**Figure 7:**
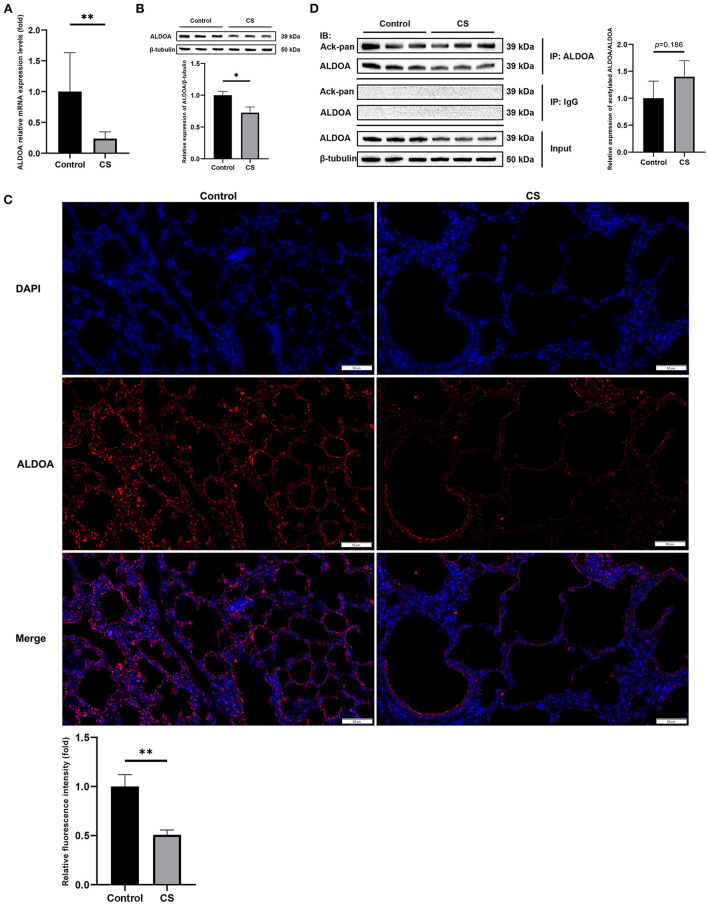
*In vivo* experimental verification of *ALDOA*. **(A)** The mRNA level of *ALDOA* in COPD mice lung tissue determined by qRT-PCR. **(B)** The protein expression level of *ALDOA* in lung tissue of mice with COPD determined and quantified by Western blot analysis. **(C)** IF staining of lung of COPD mice at 200× magnification, and the density quantified with Image J. **(D)** The acetylation level of *ALDOA* in lung tissue of mice with COPD determined and quantified by IP. *P < 0.05, **P < 0.01.

The results of qRT-PCR showed that *ALODA* mRNA expression in the lung tissue of mice from the COPD group was significantly downregulated (Figure 7A). Moreover, WB showed that the protein expression level of *ALODA* was reduced in the COPD group (Figure 7B). Consistently, IF showed that fluorescence staining of *ALDOA* in the COPD group was lower than that in the control group (Figure 7C). These results indicated that *ALDOA* was downregulated in the lung tissue of COPD mice. In addition, the calculation of the ratio of gray values of acetylated *ALDOA* and *ALDOA* by IP showed that the overall acetylation level of *ALDOA* in the lung tissue of COPD mice was increased, but not significant (*p* = 0.186; Figure 7D).

## 4. Discussion

The results of lung function measurement, H&E staining, and MLI showed that the model was reliable and credible. Although 2,479 differential genes and 564 differential proteins were defined, only 161 of these were differentially expressed at both the transcriptional and protein levels with a consistent trend, and the transcriptome and proteome were moderately correlated (*R* = 0.53). This phenomenon indicated that there were some potential regulatory mechanisms that led to an incomplete correspondence between transcription and protein expression, while the associative analysis was meaningful.

Transcriptomics and proteomics associative analysis showed that the differential expression of COPD mice induced by cigarette smoke was enriched in pathways related to the mitochondrion, energy metabolism, inflammation, immune regulation, and skeletal muscle. These pathways were closely related to COPD and extrapulmonary injury (42–46).

Our acetylomics data showed that the proteins in the lung tissue of COPD mice were mainly hyperacetylated and enriched in the pathways related to energy metabolism, mitochondrial function, ECM, and cell proliferation, and the differential acetylation sites were mainly distributed in the protein domains, such as laminin, EGF, and acyl-CoA. Laminin is a kind of non-collagenous glycoprotein, and polymerization of laminin is a key step in the basement membrane assembly (47). Basement membranes are ECMs for cell adhesion and can regulate the differentiation of cells and the maintenance of tissue structure (48). ECM is closely related to COPD, and ECM disorder can lead to lung tissue remodeling and aggravate and advance disease progression (49). The main components of the basement membrane include laminins, proteoglycans, and collagens (especially collagen IV), and EGF can stimulate the synthesis and secretion of collagen and regulate fibrin. Interestingly, we also found significant upregulation of acetylation modification of collagens, such as *COL4A2, COL6A1*, and *COL6A2*, in our acetylomics data and also enriched Pl3k-Akt signaling pathway through KEGG pathway enrichment. One study showed that the degradation and formation of collagen increased during the exacerbation of COPD (50). Another study revealed that collagen and laminin promote the proliferation, migration, and adhesion of airway smooth muscle cells in rats with COPD by upregulating the Pl3k-Akt signaling pathway (51). In addition, our data also identified the high acetylation modification of *FGG* (fibrinogen), which has the potential to predict the risk of COPD (52). These studies showed that ECMs were involved in the process of COPD, but whether it was related to their acetylation difference needs to be investigated further. Acyl-CoA dehydrogenase short chain (ACADS) may be a risk factor for COPD (53). Short chain acyl-CoA dehydrogenase (SCAD) is encoded by ACADS, and ACADS gene variants cause the mutation of SCAD, leading to mitochondrial damage and excessive production of reactive oxygen species (ROS) (54, 55). Our acetylomics data showed significant differences in acetylation modification in ACADS and ACADSB, but not in acetyl-CoA oxidase (ACOX); similarly, no study has yet assessed the correlation between ACOX and COPD, indicating that the differential acetylation of ACADS rather than ACOX may be involved in COPD.

The two most significant pathways enriched by proteomics and acetylomics associative analysis were oxidative phosphorylation (OXPHOS) and fatty acid degradation, both closely related to the function of the mitochondria. Several studies have proved that smoking-induced mitochondrial damage is one of the major mechanisms of COPD. The main features of mitochondrial damage are a decrease in the membrane potential and ATP production, excessive production of ROS, and decreased superoxide dismutase 2 (SOD2) in the mitochondria, which can cause inflammatory infiltration, damage to mitophagy, cell aging, and apoptosis (56–58). The mitochondrial dysfunction also reduced epithelial repair and corticosteroid responsiveness in the lung epithelium (44). Inflammatory mediators and ROS can also affect the structure and function of mitochondria sequentially in a vicious circle (59). Some studies showed that mitochondria DNA (mtDNA) in plasma and urine is associated with the severity and clinical phenotype of COPD (60, 61).

OXPHOS is composed of complexes I–V (CI–CV), which can form supercomplexes (SCs), such as the SC I+III_2_+IV, respirasome (62). Complex I (NADH-ubiquinone oxidoreductase) is essential for OXPHOS, participates in electron transfer and membrane potential generation, and provides electrons for respiration and ATP synthesis (63). Complex II consists of four subunits of SDHA-D, and studies have shown that complex II can directly and indirectly (during the reverse electron transfer [RET] through CI) participate in the generation of ROS (64–66); however, some other studies have shown that the loss of CII function increases the ROS (67, 68). Complex III (ubiquinol-cytochrome c oxidoreductase) also produces ROS (69). Most forms of SCs contain CIII, which, in turn, reduces the ROS formation at CI (62, 70), while the functional defect of CIII can reduce the stability of CI, CIV, and SCs and is related to the increase in ROS (71). The regulatory relationship between CIII and ROS is inconsistent, similar to CII. A previous study showed that nicotine inhibits myofibroblast through CIII with increased MitoROS, resulting in dysregulated repair during injurious responses (72). Complex IV (cytochrome c oxidase, COX) is the regulatory center of OXPHOS that can transfer reducing equivalents derived from CI or CII to free energy of oxidation through CIII and release electrochemical potential. This energy is used by the ATP synthase (CV) to synthesize ATP, and in turn, OXPHOS will be inhibited at a high ATP/ADP ratio (73). Moreover, COX is regulated by hypoxia (74) and isoforms of supernumerary subunits (75). Cloonan et al. showed that sustained expression of IRP2 increased the activity and expression of COX in the lung of mice, which led to mitochondrial dysfunction and the formation of COPD; thus, COX may be decisive for the regulation of mitochondrial iron in response to CS (76). In addition, complexes of the ETC also have different regulatory effects in immunity (77). Our proteomics and acetylomics associative analysis revealed the extensive differential expression of OXPHOS (mainly downregulation) and the differentially expressed acetylation modification in CI (*NDUFS1, NDUFA5*, and *ACAD9*), CII (SDHA), CIII (*UQCRB* and *UQCRC1*), CIV (*COX5B* and *COX6B1*), CV (*ATP5F1, ATP5F1A*, and *ATP5F1D*), and ATP synthase inhibitor, such as *ATP5IF1*. This finding suggested that the differential acetylation modification might play a critical role in the lung injury of COPD induced by cigarette smoke, although most of the OXPHOS-related proteins were not differentially expressed in our transcriptome data.

Fatty acid degradation is also closely related to mitochondria, and activated fatty acids can only be oxidized inside mitochondria and generate acetyl-coenzyme A. COPD can be accompanied by an imbalance of fatty acid metabolism in the lung, and metabolic reprogramming can also provide a feedback loop and regulate the progress of COPD; for example, by promoting inflammation and airway smooth muscle cell (ASMC) hyperplasia (43, 45, 78). Our proteomics and acetylomics associative analysis found extensive differential acetylation modification in the fatty acid degradation pathway, indicating that protein acetylation modification contributes to the interaction between fatty acid degradation and COPD. Among these, *ENO3* and *PFKM* were differentially expressed in the three levels of transcription, protein, and acetylation. Nonetheless, we did not find any relevant study on the correlation between these two genes and COPD, although one study showed that *ENO3* might be involved in lung injury caused by zinc chloride smoke (79).

Briefly, cigarette smoke can cause differential acetylation modification of proteins related to OXPHOS and fatty acid degradation, damage the electron transmission of the respiratory chain, and synthesize ATP, leading to the dysfunction of mitochondria of lung cells and promoting the formation and progress of COPD through a series of mechanisms as described earlier. Moreover, Zhang et al. and Guan et al. showed that *SIRT3* and *SIRT1* regulate the function of mitochondria of lung cells and inhibit oxidative stress, cell aging, apoptosis, and airway remodeling to reduce the lung injury of COPD induced by cigarette smoke (80, 81). Thus, regulating the acetylation modification sites of OXPHOS and fatty acid degradation-related proteins in this study may become a potential method of treating COPD.

Aldolase A (*ALDOA*) is a key enzyme of glycolysis, which can regulate metabolism and proliferation and is associated with the progression, immune infiltration, and prognosis of a variety of cancers, including lung cancer (82–84). *ALDOA* may be a biomarker to distinguish between lung cancer and COPD (85). Bai et al. revealed that *ALDOA* could limit mitochondrial autophagy and maintain NLRP3 inflammasome activity by controlling AMPK activation, leading to mitochondrial damage and inflammatory infiltration (86). This study showed that *ALDOA* was downregulated in the lung tissue of COPD; this might be a feedback regulation mechanism for mitochondrial damage and inflammation caused by cigarette smoke. Concurrently, both the omics data and the experimental verification results showed that the downregulation of *ALDOA* protein was not as obvious as at the transcriptional level, which might be insufficient to offset the inflammation and mitochondrial damage in the lung of COPD. Our acetylomics data showed that four lysine sites of *ALDOA* in the lung tissue of COPD mice were significantly hyperacetylated (147 K, log2 = 0.7338; 230 K, log2 = 0.5499; 14 K, log2 = 0.4957; 42 K, log2 = 0.4478). Consistently, IP showed that the proportion of acetylated *ALDOA* in the lung tissue of COPD mice was increased, although the results were not statistically significant. The phenomenon is not contradictory, and IP reflects the overall acetylation level of protein but cannot accurately measure the acetylation modification of each lysine site as LC-MS/MS. Nonetheless, hyperacetylation may affect the expression, structure, and function of *ALDOA*, which needs further exploration. Zhou et al. study showed that *SIRT2* inhibition promoted the protein degradation of *ALDOA* (87), which supported our hypothesis, although whether this regulation was caused by the hyperacetylation of *ALDOA* directly caused by *SIRT2* inhibition is yet to be clarified.

Nevertheless, the present study has some limitations. Multi-omics associative analyses of data of transcriptomics, proteomics, acetylomics, and scRNA-seq verified the differential expression of *ALDOA* in the lung tissue of COPD mice *via in vivo* qRT-PCR, WB, IF, and IP; however, the *in vitro* experiments are yet lacking. Therefore, an in-depth insight into the molecular and pathway mechanisms of COPD is required in the near future.

## 5. Conclusion

We carried out the multi-omics associative analysis of transcriptomics, proteomics, and acetylomics. The results proved that protein acetylation modification plays a critical role in the lungs of COPD mice and is mainly related to mitochondrial function and energy metabolism. We also identified some acetylated differentially expressed proteins related to COPD, such as *ALDOA*. Next, we preliminarily verified these genes by scRNA-seq analysis and understood their differential expression in the lung tissues of patients with COPD. Finally, we conducted an *in vivo* verification of the *ALDOA* expression in the lung tissue of COPD mice. These results suggested that the downregulation and hyperacetylation of *ALDOA* may be breakthrough points in the study of COPD. Moreover, our study showed that gene transcription and protein are not simple correspondences. Although there were some limitations, this study verified some previous findings and obtained new results. This finding indicated that multi-omics associative analysis has a unique efficacy in generating a new understanding of the classical mechanisms of diseases and identifying novel potential diagnostic and therapeutic targets through different data integration methods from traditional bioinformatics.

## Data availability statement

The datasets presented in this study can be found in online repositories. The names of the repository/repositories and accession number(s) can be found in the article/ Supplementary material.

## Ethics statement

The animal study was reviewed and approved by the Ethics Committee of the Clinical Medical College of Yangzhou University.

## Author contributions

JGa, HL, and XW designed the research and edited and revised the manuscript. JGa, LW, and JGu performed the experiments. ZY and YL analyzed the scRNA-seq data. JGa interpreted results of experiments and scRNA-seq analysis. JGa and YW prepared the figures and supplementary tables. JGa drafted the manuscript. JY and ZC participated in discussions. LM and YS approved the final version of the manuscript. All authors contributed to the article and approved the submitted version.
